# PREGnancy, HEART Health and Cardiovascular Disease (PREG-HEART): protocol for a national, direct-to-participant prospective cohort study

**DOI:** 10.1136/bmjopen-2026-120202

**Published:** 2026-10-27

**Authors:** Kathryn Hunt, Rachel Buchan, Claire Sheppard, Rachel Cartwright, Simon Fisher, Rebecca Jarman, Rebecca M Reynolds, James S Ware, Timothy James Ainsley Chico, Deborah A Lawlor, Antonio de Marvao, Upasana Tayal, UK Maternal Cardiovascular Health Collaborative Group

**Affiliations:** 1 Centre for Cardiovascular Science, Institute for Neuroscience and Cardiovascular Research, University of Edinburgh, Edinburgh, UK; 2 Medical Research Council Laboratory of Medical Sciences, Imperial College London, London, UK; 3 Royal Brompton and Harefield Hospitals, Guy’s and St Thomas’ NHS Foundation Trust, London, UK; 4 Patient Representative, London, UK; 5 National Heart and Lung Institute, Imperial College London, London, UK; 6 NIHR-BHF Cardiovascular Partnership, University of Oxford, Oxford, UK; 7 Guy's and St Thomas’ NHS Foundation Trust, London, UK; 8 British Heart Foundation Data Science Centre, Health Data Research UK, London, UK; 9 NIHR Sheffield Biomedical Research Centre, Sheffield Teaching Hospitals NHS Foundation Trust, The University of Sheffield, Sheffield, UK; 10 School of Medicine and Population Health, University of Sheffield, Sheffield, UK; 11 Medical Research Council Integrative Epidemiology Unit, University of Bristol, Bristol, UK; 12 Population Health Science, Bristol Medical School, University of Bristol, Bristol, UK; 13 Department of Women and Children’s Health, School of Life Course & Population Sciences, King’s College London, London, UK; 14 British Heart Foundation Centre of Research Excellence, School of Cardiovascular and Metabolic Medicine & Sciences, King’s College London, London, UK

**Keywords:** Pregnancy, Rare Diseases, Heart failure, Genetics

## Abstract

**Abstract:**

**Introduction:**

Cardiovascular disease is a leading cause of maternal and neonatal morbidity and mortality in the UK. Its prevalence in pregnancy continues to rise, driven by both improved survival of women with congenital and inherited heart disease into reproductive age and an increasing burden of acquired cardiovascular risk factors. However, its natural history and optimal management remain poorly defined. Current research is limited by small sample sizes, drawn from highly selected patient cohorts from individual units. The aim of the PREGnancy, HEART Health and Cardiovascular Disease (PREG-HEART) study is to develop a patient-driven, clinically relevant digital platform to understand the epidemiology of cardiovascular disease in pregnancy and support clinical trials of management strategies. This paper provides the protocol for PREG-HEART which will start with a 6-month pilot study.

**Methods and analysis:**

PREG-HEART will use an online, direct-to-patient platform to enrol patients with cardiovascular disease in pregnancy alongside healthy pregnant controls. Enrolled women will be invited to provide self-reported demographic and clinical data and consent to linkage with national health records for long-term follow-up. We will also seek consent for storage and analysis of leftover clinical biosamples and to re-contact participants, enabling recruitment into sub-studies and clinical trials.

Planned analysis for the pilot study at 6 months will assess feasibility, including recruitment rates, case-mix of cardiovascular diagnoses, and participant geographical, socio-economic and ethnic background, compared with the UK general pregnant population. Findings from the pilot study will inform subsequent phases of PREG-HEART, which will explore associations between different cardiovascular diagnoses and adverse cardiovascular, obstetric and neonatal events. We will work closely with patients and clinicians to define priority research questions and use the PREG-HEART platform to support a range of observational and interventional studies to address these.

**Ethics and dissemination:**

Approved by the West Midlands Solihull Research Ethics Committee (IRAS 349694; REC 25/WM/0142). Findings will be returned directly to participants, disseminated to clinicians through professional societies and the National Institute for Health and Care Research-British Heart Foundation Cardiovascular Partnership, and to the public through partner charities, as well as by peer-reviewed publication.

**Trial registration number:**

ISRCTN11700499.

**Strengths and limitations of this study**

- A national, direct-to-participant design allows enrolment independent of geography or clinical centre, avoiding the institutional selection bias of clinician-submitted registries.
- Contemporaneous recruitment of healthy pregnant controls allows comparison within the same recruitment framework and time period.
- Consent is obtained at enrolment for lifetime linkage to national health records, for retention of residual clinical biosamples and for recontact, enabling longitudinal, mechanistic and interventional studies.
- Enrolment currently requires Internet access and written English, which may limit participation by some under-served groups, with translated materials planned as the platform expands.
- The broad eligibility criteria span heterogeneous cardiovascular conditions, so numbers within individual diagnostic groups will be small in the early phases.

## Introduction

 Cardiovascular disease is a leading cause of maternal morbidity and mortality in the UK and a major contributor to adverse fetal and neonatal outcomes.^1–3^ Up to 4% of pregnant women have cardiovascular disease and this prevalence is increasing, driven both by improved survival into adulthood of women with inherited and congenital heart diseases and by the rising burden of acquired risk factors for cardiovascular disease, such as advanced maternal age, obesity, diabetes mellitus and hypertension.^4^

There has been no significant improvement in maternal death rates from cardiovascular disease over recent decades and outcomes in the UK compare poorly to other high-income countries.^1 5^ Moreover, there is stark inequality in women’s maternal health outcomes in the UK, with women of Black ethnicity nearly three times more likely than White women to die in childbirth and Asian women almost twice as likely.^1^ Maternal mortality is also twice as high in the most socio-economically deprived areas of the UK compared with the most affluent.^1^

Although the cumulative incidence of cardiovascular disease in pregnancy is rising, the relative rarity of individual diagnoses limits research into specific conditions in pregnancy. The natural history of cardiovascular diseases in pregnancy, institutional and regional variation in management and the relative contributions of genetic and environmental factors remain poorly characterised, largely because sufficiently large cohorts are difficult to assemble. Interventional trials are typically small, underpowered and restricted to highly selected patient groups.^4^

National data collection on cardiovascular disease in pregnancy has up to now relied on clinicians to identify patients and submit data to centralised registries. Such an approach is limited, as it places pressure on busy clinicians, is biased towards better-resourced units and restricts opportunities for participation. For example, the Registry Of Pregnancy And Cardiac disease (ROPAC) study across Europe recruited fewer than 6000 women over 10 years,^6^ whereas our preliminary work identified 29 890 women entering pregnancy with previously diagnosed cardiac disease during 2019–2024 in England alone.^7^ An over-reliance on clinician-directed registries also results in marked geographical variation in research access, particularly disadvantaging women who live far from large academic centres.

These factors have direct impacts on clinical care; for example, risk stratification tools which guide patient counselling and management may be based on data which is either outdated or poorly representative of real-world populations. Regional disparities in research participation compound systemic inequities, particularly for groups historically under-represented and underserved by cardiovascular research.^8^

Maternal cardiovascular health is identified as a priority area, and in 2022 was adopted as a flagship theme by the National Institute for Health and Care Research (NIHR)-British Heart Foundation (BHF) Cardiovascular Partnership. The Partnership is a strategic initiative and NIHR Translational Research Collaboration representing 19 member centres (including 11 NIHR Biomedical Research Centres, nine BHF Centres of Research Excellence and four Scottish cardiovascular centres), that facilitates UK-wide cardiovascular research at scale.

Within this framework, the PREGnancy, HEART Health and Cardiovascular Disease (PREG-HEART) study has been conceived and designed, as a national, direct-to-patient platform. Its overarching aim is to build a large, representative cohort of patients with cardiovascular disease in pregnancy alongside healthy pregnant controls, to enable improved understanding of the contemporaneous natural history of cardiovascular disease in pregnancy, enable robust epidemiological research, and provide a scalable resource for clinical trials and biomarker discovery. By adopting a direct-to-patient approach to recruitment, we seek to overcome the geographical and socio-economic barriers which have driven the under-representation of women—particularly pregnant women—in cardiovascular research.^9^

### Study aims

The PREG-HEART study will begin as a pilot phase, which will aim to demonstrate the feasibility of direct-to-patient recruitment for studying cardiovascular disease in pregnancy. At 6 and 12 months, we will assess recruitment rates, case-mix of cardiovascular diagnoses among participants, and the sociodemographic, geographical, and ethnic background of participants to determine which, if any, groups are under-represented by our approach.

Findings from the pilot study will inform further development of the PREG-HEART platform as a patient-driven resource with the ability to establish a large, representative, heterogenous, deeply-phenotyped patient cohorts with linkage to obstetric, cardiovascular and long-term outcomes. This will help build national capacity for cardiovascular research and support observational and interventional studies to address priority research questions for patients and clinicians.

Overall study aims and objectives are summarised in box 1.

Box 1 **Aims of PREGnancy, HEART Health and Cardiovascular Disease study**

**Overarching aim:**

- To establish a large, representative and deeply-phenotyped cohort of women with cardiovascular disease in pregnancy, alongside healthy pregnant controls, to provide a national platform for collaborative clinical and translational research.

**Pilot study objectives:**

- Feasibility: demonstrate that a direct-to-patient online platform can effectively recruit participants with cardiovascular disease in pregnancy.
- Representation: assess the geographical, socio-economic and ethnic diversity of recruited participants and identify under-represented groups.

**Long-term objectives of scaled-up study:**

- Natural history and management: characterise the natural history of different forms of cardiovascular disease in pregnancy and evaluate regional and institutional variation in management practices and outcomes across the UK.
- Bioresource: create a linked bioresource of clinical samples (blood, saliva, urine) to support multiomic research, improve understanding of pathophysiological mechanisms and develop biomarkers for diagnosis and risk stratification.
- Platform for trials: overcome historic, geographical and institutional barriers to research participation by providing diverse patient groups with linked outcome data that are readily available for recruitment into clinical trials.

## Methods and analysis

### Study design

Study design and participant flow are illustrated in figure 1. An overview of the study is shown in the online supplemental graphical abstract.

**Figure 1:**
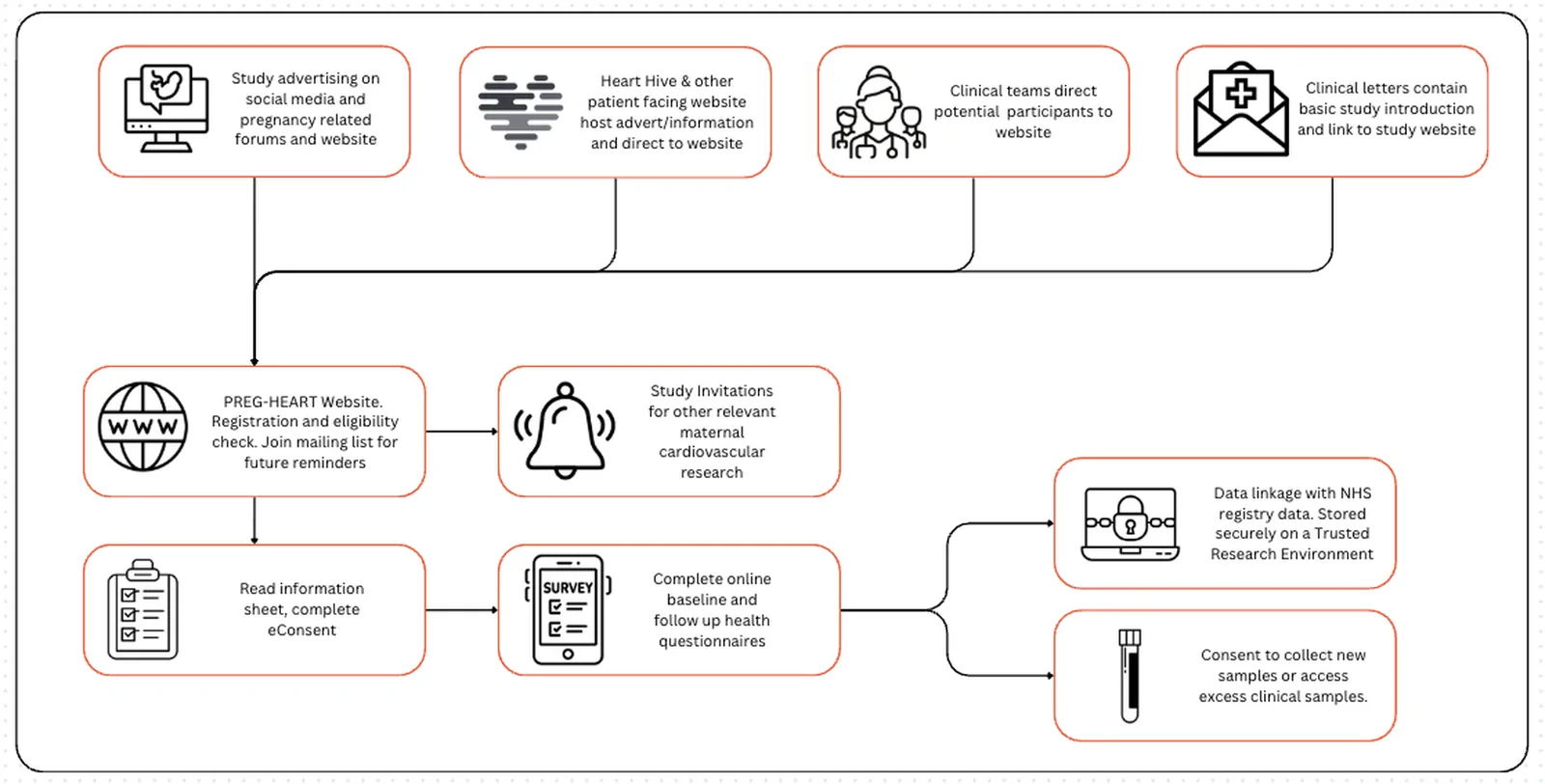
PREG-HEART study design and participant flow. NHS, National Health Service; PREG-HEART, PREGnancy, HEART Health and Cardiovascular Disease.

PREG-HEART is a bespoke patient-facing, online, observational, prospective study which will enrol participants with cardiovascular disease in pregnancy (cases) and healthy pregnant women (controls) on an ongoing basis. The study was co-designed with a patient steering group and builds on existing infrastructure of the Heart Hive (https://thehearthive.org), an established patient-facing platform for cardiomyopathy and myocarditis research.^10^

### Oversight and governance

The study is sponsored by Imperial College London. Study design and delivery are informed by a multidisciplinary working group from the Maternal Cardiovascular Health Theme of the NIHR-BHF Cardiovascular Partnership, which includes patient representatives and experts in clinical trials, data science and data governance (membership provided in online supplemental appendix 1). An executive steering committee drawn from this working group will be responsible for ongoing governance and oversight of the study.

### Funding and registration

Set-up and initial recruitment to the pilot study is funded by pump-priming funding from the NIHR-BHF Cardiovascular Partnership. PREG-HEART has been prospectively registered on the ISRCTN clinical study registry (ISRCTN11700499).

### Patients

Eligibility criteria are summarised in box 2. The study is being conducted in accordance with the principles of Good Clinical Practice and the World Medical Association Declaration of Helsinki. All participants will provide written informed consent electronically, via a step-by-step online consent process culminating in an electronic signature (online supplemental file 2). Participants are eligible from 16 years of age. Under the relevant UK legislation, participants aged 16 and 17 years who have the capacity can consent for themselves; parental assent is not sought.

Box 2 **Eligibility criteria for the PREGnancy, HEART Health and Cardiovascular Disease study**

**Inclusion criteria:**

- Aged 16 years or above.
- Capacity to provide informed consent.

For those enrolling as participants with cardiovascular disease (cases):

- Any history of pregnancy, either current or previous (with pregnancy defined as a positive pregnancy test and/or ultrasound confirmed pregnancy).
- Self-reported confirmed diagnosis of cardiovascular disease which predates pregnancy, or was newly diagnosed during pregnancy or within 6 months postpartum, including:
  - Ventricular dysfunction:
    - Cardiomyopathies (inherited or acquired, including peripartum cardiomyopathy).
    - Heart failure of any aetiology.
  - Valvular heart disease:
    - Including previous valve replacement.
  - Congenital cardiac lesions:
    - Including following surgical correction of congenital lesion.
  - Arrhythmia:
    - Cardiac conduction defects and arrhythmias.
    - History of previous cardiac ablation.
    - Inherited arrhythmia syndromes.
  - Coronary artery diseases, including:
    - Atherosclerotic disease.
    - Spontaneous coronary artery dissection.
    - Congenital coronary anomalies or arteriovenous malformations.
  - Chronic systemic hypertension.
  - Pulmonary hypertension.
  - Aortic disease.
  - Connective tissue disease:
    - Heritable collagen, elastin or glycosaminoglycan disorders.
    - Autoimmune connective tissue diseases and vasculitides.
  - Thromboembolic disease.
  - Carriers of (pathogenic) genetic variants associated with cardiovascular conditions.
  - Other relevant long-term cardiovascular diagnoses as determined by the study investigators.

For those enrolling as participants without cardiovascular disease (controls):

- Currently pregnant (with pregnancy defined as a positive pregnancy test and/or ultrasound confirmed pregnancy).
- No diagnosed cardiovascular disease, as defined by the conditions listed above.

**Exclusion criteria:**

- Patients who lack capacity to consent.
- People who do not have access to the Internet and/or who are unable to provide an email address for study correspondence.
- Although vulnerable groups (eg, prisoners, those in dependent relationships) are not ineligible to participate, clinician discretion will be advised when considering whether to signpost potential participants to the registry where such vulnerabilities may be present.

### Recruitment and consent

Eligible participants will be directed to the PREG-HEART online platform via multiple routes, including social media campaigns, patient charities and support groups, national and local media promotion, referral from clinicians in relevant specialties (obstetrics, cardiology, midwifery, anaesthetics, clinical genetics, general practice and others), and display of study information to female participants in the Heart Hive registry. Clinician awareness is raised through the NIHR-BHF Cardiovascular Partnership and national professional societies, using newsletters, meetings and printed materials for clinical and community settings, alongside charity partners with reach into under-represented groups. Recruitment routes are kept under review and adapted as the study develops.

On accessing the online platform (https://thehearthive.org/preg-heart), potential participants will complete screening questions to confirm eligibility. A short plain-English summary of the study and of what taking part involves appears on the study landing page and in all recruitment materials. Those wishing to proceed enter a 10-screen electronic consent walkthrough, in which the information is presented in short sections with supporting graphics and comprehension checks, culminating in the consent form (online supplemental file 2). The full participant information sheet as approved by the Research Ethics Committee (online supplemental file 1) can be read, downloaded or printed at any point before, during or after consent, and the study team can be contacted by email at any stage.

Participant information materials (provided in online supplemental file 1) will explain the use of all data and acknowledge that participants can opt out of completing any questions or providing consent for data linkage or storage and use of leftover blood from clinical samples. It also explains how to withdraw from the study should they wish.

### Study procedures

#### Baseline evaluations

On registration, participants will be requested to enter socio-demographic details and health data relevant to their cardiovascular diagnosis and current/previous pregnancies via an online questionnaire. Data collected includes socio-demographic and lifestyle information, medical, surgical and obstetric history, family history of cardiac disease, medications, ECG, echocardiography, cardiac MRI and other investigations participants may undergo as part of their clinical care. The baseline questionnaire we will use for data collection is included in online supplemental appendix 2.

To improve completeness and accuracy of collected clinical data and reduce burden on participants associated with data entry, we will request that participants send (to a secure National Health Service (NHS) study email address) copies of clinic letters and results of imaging, genetic, biochemical or histopathological investigations which pertain to their cardiovascular diagnosis or pregnancy, where these are available. These documents will be securely uploaded to the PREG-HEART platform by the study team.

#### Follow-up and long-term data linkage

In addition to the online questionnaire completed when participants first register on the PREG-HEART platform, we will offer participants an additional questionnaire within 6 months of the expected pregnancy due date. This will collect information about obstetric and neonatal outcomes. The follow-up questionnaire we will use for data collection is included in online supplemental appendix 3.

Consent will also be sought for collection of follow-up data over their lifetime from NHS digital and national registries (eg, Office for National Statistics, Hospital Episode Statistics, Patient Episode Dataset for Wales, Scottish Morbidity Record (SMR02), Diagnostic Imaging Dataset, Maternity Services Data Set, Maternity Indicators Dataset, Scottish Birth Record, National Neonatal Research Database, Intensive Care National Audit and Research Centre, and equivalent national registries, as well as further linkage to datasets through the BHF Data Science Centre www.bhfdatasciencecentre.org/for-researchers/datasets and Clinical Practice Research Datalink https://www.cprd.com) and from medical records (eg, primary care data via Vision www.visionhealth.co.uk, Emis www.emishealth.com, SystmOne www.tpp-uk.com/products/systmone, maternity and neonatal data via BadgerNet Maternity www.systemc.com/our-solutions/healthcare/maternity-neonatal, K2 www.k2ms.com, TrakCare for Maternity www.intersystems.com/uk/products/trakcare, and equivalent electronic health and maternity care records). This data linkage aspect of the PREG-HEART study is dependent on future funding.

#### Biosamples

Consent will be sought from participants to collect and store leftover blood from routinely collected clinical samples in pregnancy (which would otherwise be discarded), for unspecified future research use. We will also seek consent to recontact participants in future to invite them to provide additional biosamples (blood, saliva and/or urine).

In the initial phase of this study, existing local biobank infrastructure will be used for processing and storage of blood leftover from routinely collected clinical samples. In future phases of the study and as additional funding is secured, biosamples will be transported to a central secure facility for processing and storage.

Collected biosamples may undergo genomic, transcriptomic, proteomic, lipidomic, metabolomic and biochemical analyses, dependent on future funding. Participants provide consent for sequencing up to the level of the whole genome. Data generated from the samples will be linked with demographic and clinical data.

### Substudies

Participants will be asked to provide their consent to be contacted regarding participating in additional ethically approved research studies, focusing on specific aspects of cardiovascular disease in pregnancy. These studies may be led by Imperial College London or collaborating academic and healthcare centres. Specific consent would be obtained from patients for these additional studies, following a clear explanation of any risks involved.

### Data flow and management

Consent forms and data collected in study questionnaires will be entered via the Heart Hive, a secure online web portal built in collaboration with the Broad Clinical Labs (a subsidiary of the Broad Institute, a not-for-profit research institute) on the Juniper data platform. This data will be stored, along with copies of clinic letters and investigation reports submitted by participants, on secure servers in accordance with a data processing agreement with Imperial College London (the data controller).

Additional funding will be sought to enable data linkage with NHS digital and national registries, allowing incorporation of additional patient-level information from maternity services, primary care, community dispensing, hospital episode and death statistics. Linked data will be stored in an accredited Trusted Research Environment in collaboration with the BHF Data Science Centre. The BHF Data Science Centre is part of Health Data Research UK, the UK’s institute for health data science, and will support coordinated data storage, access, sharing, analysis and linkage in line with best practices in data management, security and governance.

#### Outcome measures

The primary outcome is the feasibility of direct-to-participant recruitment to a cohort study of cardiovascular disease in pregnancy, assessed over the initial 6-month pilot period by the number of participants enrolled, the baseline questionnaire completion rate, the proportion consenting to data linkage, biosample retention and recontact, participant flow through each stage of the enrolment pathway, and the case mix of self-reported cardiovascular diagnoses compared with estimated UK prevalences.

Secondary outcomes are the geographical, socio-economic and ethnic representativeness of the enrolled cohort compared with the UK pregnant population and, in subsequent phases, adverse cardiovascular, obstetric and neonatal outcomes ascertained through follow-up questionnaires and health record linkage.

### Data analysis

#### Pilot study

We will assess recruitment to the PREG-HEART platform at 6 months and report the outcomes above. We will also report participant flow through each stage of enrolment, including visits to the study landing page, completion of the eligibility screen, progression to the participant information and consent process, and completion of consent and of the baseline questionnaire, so that the points at which potential participants are lost can be identified and addressed.

We will compare participants’ self-reported cardiovascular diagnoses to estimated UK prevalences. Our group estimates the UK prevalence of heart disease pre-dating pregnancy to be at least 1% (20 475 out of 1 967 250 pregnancies in England between 1 November 2019 and 30 June 2024).^7^ Of these, 1520 women had heart failure and 730 had cardiomyopathy, identified using electronic health record coding data. Based on these figures, we would consider enrolling 25 participants with heart failure or cardiomyopathy (10% of cases over 6 months) during the pilot phase as evidence of feasibility, within an anticipated total of approximately 200 participants with cardiovascular disease over the same period.

We will report the geographical, socio-economic and ethnic diversity of our enrolled cohort with comparison to that of the UK general pregnant population. This will allow us to identify under-represented groups and thus will inform our approaches to recruitment for the larger scaled-up study.

#### Future work

Alongside future scale-up of the PREG-HEART platform, we will report outcome data for the cohort collected through follow-up questionnaires and data linkage on an approximately annual basis. Reported outcomes will include the incidence of major adverse cardiovascular events, including cardiovascular death, arrhythmic and heart failure events, stroke and atrial fibrillation. Adverse obstetric and neonatal outcomes reported will include maternal death, stillbirth or neonatal death, congenital anomaly, preterm delivery, fetal growth restriction, neonatal unit admission, antenatal or postpartum haemorrhage and maternal hypertensive disease. Outcomes will be stratified according to participant socio-demographic status and ethnicity, and regional and institutional variations in outcomes will be reported.

#### Patient and public involvement

Women with lived experience of cardiovascular disease in pregnancy have been involved as project partners in all aspects of the study from inception, including setting research questions, study design, protocol development, design of patient surveys and all other patient facing materials, development and testing of the online platform, co-authorship of this paper and planning the dissemination strategy for the registry.

A dedicated patient advisory group, including women with lived experience of cardiovascular disease in pregnancy, will provide ongoing input into strategies for recruitment, further development of the registry, data analysis, dissemination of findings and the development of additional participant-facing materials such as a video participant information sheet.

All patient partners are compensated for their time in line with NIHR feasibility guidance.

## Ethics and dissemination

This study involves human participants and was approved by the West Midlands Solihull Research Ethics Committee (IRAS project ID: 349694; REC reference: 25/WM/0142).

Results from the PREG-HEART study will be presented at conferences, published in peer-reviewed academic journals and be made available on the study’s website (https://thehearthive.org/preg-heart). Findings will also be reported on the ISRCTN registry record and returned directly to participants through the study platform. Dissemination to clinicians and researchers will be through national professional societies and the NIHR-BHF Cardiovascular Partnership and to the public through partner charities and patient organisations.

## Discussion

PREG-HEART is designed to address structural barriers that have limited evidence generation in cardiovascular disease in pregnancy. Individual cardiovascular conditions are uncommon within any single maternity service while existing registries have largely relied on clinicians to identify participants and submit data. This places additional demands on busy clinical teams, favours recruitment from better-resourced specialist centres and may limit research access for women who live or receive care elsewhere. PREG-HEART uses a national, direct-to-participant approach, enabling women to enrol irrespective of where they live or receive care and providing a common platform for participation across the UK.

The approach has potential limitations that require evaluation. Participation currently requires Internet access, an email address and sufficient written English, which may disadvantage groups already under-represented in cardiovascular research. Cardiovascular diagnoses are initially participant-reported and the broad eligibility criteria encompass heterogeneous conditions, meaning that numbers within individual diagnostic groups will be relatively small during the early phases. We will therefore assess the geographical, socio-economic, ethnic and diagnostic distribution of participants against the wider UK pregnant population and examine participant progression through the enrolment pathway. This will identify gaps in representation and inform adaptation of recruitment strategies, translated and accessible materials, community engagement and supported enrolment as the platform develops.

The value of PREG-HEART extends beyond the establishment of an observational cohort. For patients, it provides a route into cardiovascular research that does not depend on attendance at a specialist academic centre, while consent for recontact provides opportunities to participate in future observational and interventional studies. Participant-reported follow-up can also capture symptoms, functional status, treatment burden and other outcomes that may not be available in routinely collected health data. For clinicians and health services, longitudinal linkage of pregnancy, cardiovascular and neonatal outcomes will enable variation in management, care pathways and outcomes across regions and institutions to be examined.

For researchers, the combination of a consented longitudinal cohort, routine health-data linkage, recontact and future biosample collection provides infrastructure that individual studies would otherwise need to establish independently. The platform can support epidemiological and mechanistic studies, biomarker and risk-prediction research and recruitment to future clinical trials. Patients have been involved in the development of PREG-HEART from inception, including study design, participant-facing materials, platform development, analysis and dissemination, and will continue to help shape research priorities and outputs. As the cohort grows, PREG-HEART has the potential to provide an enduring national resource for collaborative maternal cardiovascular research, strengthening the evidence base and supporting the evaluation and translation of new approaches to prevention, diagnosis and management.

## Supplementary material

10.1136/bmjopen-2026-120202 online supplemental file 1

10.1136/bmjopen-2026-120202 online supplemental file 2

10.1136/bmjopen-2026-120202 online supplemental file 3

10.1136/bmjopen-2026-120202 online supplemental file 4
