# Supplementary material for: PREGnancy, HEART Health and Cardiovascular Disease (PREG-HEART): protocol for a national, direct-to-participant prospective cohort study
Source: BMJ Open. 2026 Oct 27;16(10):e120202. doi: 10.1136/bmjopen-2026-120202 (PMC13723820; doi:10.1136/bmjopen-2026-120202)
Supplement: Supplementary file 1 [file bmjopen-2026-120202.reviewer_comments.pdf]

PEER REVIEW HISTORY

BMJ Open publishes all reviews undertaken for accepted manuscripts. Reviewers are asked to complete a checklist review form and are provided with free text boxes to elaborate on their assessment. These free text comments are reproduced below.

ARTICLE DETAILS

Title (Provisional)

PREGnancy, HEART Health and Cardiovascular Disease (PREG-HEART): protocol for a national, direct-to-participant prospective cohort study

Authors

Hunt, Kathryn; Buchan, Rachel; Collaborative Group, UK Maternal Cardiovascular Health; Sheppard, Claire; Cartwright, Rachel; Fisher, Simon; Jarman, Rebecca; Reynolds, Rebecca M; Ware, James S; Chico, Timothy James; Lawlor, Deborah A; de Marvao, Antonio; Tayal, Upasana

---

VERSION 1 - REVIEW

**Reviewer**	**1**
**Name**	**Lindley, Richard**
**Affiliation**	**University of Sydney, Medicine**
**Date**	**29-Jun-2026**
**COI**	**None**

This pilot registry addresses an important aspect of women's public health in the UK. An inception cohort of such women, and controls, would provide a very valuable research resource that will have the potential to reduce future CVD mortality and morbidity in women who have been pregnant.

The researchers have designed a recruitment strategy to reduce the selection bias of current registries. However, I do note that the current information leaflet is very long.

1) Are you able to provide a summary sheet for potential participants.

2) The inclusion of the Imperial College Privacy Notices is not welcome at this stage of the project - surely not in the patient's interest to have this legalese forced on them at this stage. Why can you not provide a short summary of this privacy notice? Maybe something like, "we are required to provide you with information regarding how we manage your date. This will be done with our highest standards of security to ensure your data remains private, and only used for the medical research purposes we have described. Please click this link for the details of how we will do this."

As you know, most people do not read legal notices - how many of the authors have actually read the agreement for the latest update for your iPhone or other software, or read the actual legal contract for your airfare? The double standard here in everyday life remains extraordinary.

3) I wonder if your patient partners are completely happy with the length and detail of the information leaflet? Could you also provide shorter summaries, and perhaps a YouTube information video to supplement information provision?

4) There is no mention of being able to recruit people whose main language is not English, and it would be worth discussing if the commonest alternate languages could be incorporated into the information leaflets and website. With modern technology, I would hope you could help such people be included.

5) In Figure 1, you show your initial recruitment strategy. It would be useful for you to add exactly how you will educate such clinical teams to direct potential participants to the website, and how you could snowball this and other recruitment strategies, perhaps with the help of the community pregnancy groups and antenatal groups?

6) In your pilot feasibility, you should collect how many individual visits are made to the initial website page, and then record the drop off for say, reading (or downloading) the information leaflet, and then signing the consent form.

7) I note that your patient partners will receive appropriate approved remuneration but will you be able to offer a modest reward for the women you consent and provide follow-up data in the registry?

8) It would be nice to acknowledge all your collaboration in the authorship. How about the authorship is your "PREG-HEART Collaborative Group", and you then list the writing committee in the appendix? This, of course, will depend on the BMJ Open guidelines, but the entire collaboration will have contributed to this idea.

VERSION 1 - AUTHOR RESPONSE

Response to Reviewer 1

General comment: This pilot registry addresses an important aspect of women's public health in the UK. An inception cohort of such women, and controls, would provide a very valuable research resource that will have the potential to reduce future CVD mortality and morbidity in women who have been pregnant. The researchers have designed a recruitment strategy to reduce the selection bias of current registries.

Response

We thank the reviewer for this positive assessment and for recognising the potential of PREG-HEART as a research resource.

Comment 1: However, I do note that the current information leaflet is very long. Are you able to provide a summary sheet for potential participants.

Response

Supplementary File 1 is the full participant information sheet as approved by the West Midlands - Solihull Research Ethics Committee (IRAS 349694; REC reference 25/WM/0142), and the study has been recruiting on this basis since February 2026. Much of its length reflects content added at the request of the REC and the sponsor during the ethical review process, and the HRA requires that the complete document is available to participants. It is therefore not a document the authors are able to revise in response to journal peer review.

It is not, however, presented to participants as a single document. Information is presented in layered form: a short plain-English summary on the study landing page, followed by a ten-screen electronic consent walkthrough presenting the information in short sections with comprehension checks, with the full sheet available to read, download or print at any point. We accept this was not clear in the original manuscript and have revised the Methods accordingly. We have also added the eConsent screens as Supplementary File 2 so that readers can see the information as participants see it.

These materials reflect what was approved and funded for the first phase of the study. The next phase of PREG-HEART, for which funding has been sought, includes a costed programme to develop supplementary participant materials, including a short summary sheet and a video participant information sheet with audio narration, together with telephone and email support through enrolment for women who would prefer assistance. Any new participant-facing materials will be submitted to the REC for approval before use.

We have edited "Methods, Recruitment and consent", paragraph 2 as follows:

"~~Those eligible will be provided with detailed participant information online and will complete a step-by-step electronic consent process. This includes multi-page walkthroughs and comprehension checks to ensure understanding before enrolment.~~ **A short plain-English summary of the study and of what taking part involves appears on the study landing page and in all recruitment materials. Those wishing to proceed enter a ten-screen electronic consent walkthrough, in which the information is presented in short sections with supporting graphics and comprehension checks, culminating in the consent form (Supplementary File 2). The full participant information sheet as approved by the Research Ethics Committee (Supplementary File 1) can be read, downloaded or printed at any point before, during or after consent, and the study team can be contacted by email at any stage."**

Comment 2: The inclusion of the Imperial College Privacy Notices is not welcome at this stage of the project - surely not in the patient's interest to have this legalese forced on them at this stage. Why can you not provide a short summary of this privacy notice? Maybe something like, "we are required to provide you with information regarding how we manage your date. This will be done with our highest standards of security to ensure your data remains private, and only used for the medical research purposes we have described. Please click this link for the details of how we will do this." As you know, most people do not read legal notices - how many of the authors have actually read the agreement for the latest update for your iPhone or other software, or read the actual legal contract for your airfare? The double standard here in everyday life remains extraordinary.

Response

We agree with the general point, and recognise the wider phenomenon the reviewer describes. However, the wording is not at the authors' discretion. It follows the HRA's standard transparency wording, which is required by Imperial College London as sponsor and data controller. As above, it forms part of the REC-approved documentation. Participants do experience something close to what the reviewer proposes: data use is summarised in plain English within the eConsent walkthrough, with the full notice available by link and download.

Comment 3: I wonder if your patient partners are completely happy with the length and detail of the information leaflet? Could you also provide shorter summaries, and perhaps a YouTube information video to supplement information provision?

Response

We agree and are grateful for the suggestion.

Patient partners have been involved from inception, including in the design of all participant-facing materials, and also as a co-author in this protocol manuscript. As noted above, a video participant information sheet with audio narration is a costed element of the planned next phase, developed with PPIE input and intended specifically to support women who may find a long written document difficult.

We have edited "Patient and public involvement and engagement", paragraph 2 as follows:

"A dedicated patient advisory group, including women with lived experience of cardiovascular disease in pregnancy, will provide ongoing input into strategies for recruitment, further development of the registry, data analysis, and dissemination of findings, **including the development of additional participant-facing materials such as a video participant information sheet."**

Comment 4: There is no mention of being able to recruit people whose main language is not English, and it would be worth discussing if the commonest alternate languages could be incorporated into the information leaflets and website. With modern technology, I would hope you could help such people be included.

Response

We agree, and thank the reviewer for raising it. In this first phase, approved materials are in English only, as the pump-priming award did not fund certified translation, and machine translation is not appropriate for consent to lifelong data linkage and genomic research. In the second phase, translation into four languages is costed, alongside partnerships with organisations working with under-served communities. Translated materials will require REC approval before use.

PREG-HEART is intended as a long-term national resource rather than a fixed-term study, and participant-facing materials will continue to be developed as the platform grows. The protocol already specifies that the pilot will report the geographical, socio-economic and ethnic distribution of participants against the UK pregnant population, which is how this shortfall will be quantified. We have reflected the point in the new "Strengths and limitations of this study" section added at the Editorial Office's request.

"**Enrolment currently requires internet access and written English, which may limit participation by some under-served groups, with translated materials planned as the platform expands.**"

Comment 5: In Figure 1, you show your initial recruitment strategy. It would be useful for you to add exactly how you will educate such clinical teams to direct potential participants to the website, and how you could snowball this and other recruitment strategies, perhaps with the help of the community pregnancy groups and antenatal groups?

Response

We agree, and have expanded this. The manuscript described recruitment routes but not the mechanisms behind them.

Clinician awareness is built through the NIHR-BHF Cardiovascular Partnership, whose 19 member centres span the four nations, and through national professional societies, several of which have provided formal endorsement or in-kind support, including the Royal College of Midwives and the UK Maternal Cardiology Society. Engagement runs through society newsletters, conferences, webinars and social media, supported by printed materials for maternity services, cardiology clinics and GP practices. Onward reach into community and antenatal groups is through charity and community partners, including Cardiomyopathy UK, the Pumping Marvellous Foundation and organisations

working with under-served communities. Because enrolment requires only a link, participants and partner organisations can share the study directly within their own networks.

We have edited "Recruitment and consent", paragraph 1 as follows:

~~"Clinician awareness will be raised through dissemination across local and national professional networks.~~ **Clinician awareness is raised through the NIHR-BHF Cardiovascular Partnership and national professional societies, using newsletters, meetings and printed materials for clinical and community settings, alongside charity partners with reach into under-represented groups. Recruitment routes are kept under review and adapted as the study develops."**

Comment 6: In your pilot feasibility, you should collect how many individual visits are made to the initial website page, and then record the drop off for say, reading (or downloading) the information leaflet, and then signing the consent form.

Response

We agree, and these data are already collected. In the pilot phase, 161 individuals created an account and 64 went on to consent, and reducing this drop-off is a specific objective of the next phase of the study. We have added the enrolment funnel to the feasibility outcomes.

We have edited "Data analysis", *Pilot study*, paragraph 1 as follows:

"**We will assess recruitment to the PREG-HEART platform at 6 months and report the outcomes above. We will also report participant flow through each stage of enrolment, including visits to the study landing page, completion of the eligibility screen, progression to the participant information and consent process, and completion of consent and of the baseline questionnaire, so that the points at which potential participants are lost can be identified and addressed."**

Comment 7: I note that your patient partners will receive appropriate approved remuneration but will you be able to offer a modest reward for the women you consent and provide follow-up data in the registry?

Response

Payment to participants is not part of the approved protocol. Participation involves no visits, tests or procedures beyond questionnaires, and payment is not usual practice in observational registries of this kind in the UK. More fundamentally, our ambition is for PREG-HEART to become part of routine antenatal and postnatal care, offered to all

eligible women alongside NHS digital services. Per-participant payment would not be sustainable at that scale, and would work against the aim of making research participation a routine option rather than a funded exception.

Comment 8: It would be nice to acknowledge all your collaboration in the authorship. How about the authorship is your "PREG-HEART Collaborative Group", and you then list the writing committee in the appendix? This, of course, will depend on the BMJ Open guidelines, but the entire collaboration will have contributed to this idea.

Response

We agree with the principle, and the manuscript already reflects it. The named authors are those who meet all four ICMJE criteria. Appendix 1 lists the membership of the NIHR-BHF Cardiovascular Partnership Maternal Cardiovascular Health working group, within which the study was conceived and developed.
