# Supplementary material for: PREGnancy, HEART Health and Cardiovascular Disease (PREG-HEART): protocol for a national, direct-to-participant prospective cohort study
Source: BMJ Open. 2026 Oct 27;16(10):e120202. doi: 10.1136/bmjopen-2026-120202 (PMC13723820; doi:10.1136/bmjopen-2026-120202)
Supplement: Supplementary file 3 [file bmjopen-16-10-s002.docx]

**Supplementary File 2: PREG-HEART electronic consent process**

Screens presented to potential participants during online enrolment, culminating in the informed consent form.

PAGE 1

PAGE 2

PAGE 3

PAGE 4

PAGE 5

PAGE 6

PAGE 7

PAGE 8

PAGE 9

PAGE 10 – INFORMED CONSENT FORM
